# Supplementary material for: A neurocomputational theory of action regulation predicts motor behavior in neurotypical individuals and patients with Parkinson’s disease
Source: PLoS Comput Biol. 2022 Nov 17;18(11):e1010111. doi: 10.1371/journal.pcbi.1010111 (PMC9714880; doi:10.1371/journal.pcbi.1010111)
Supplement: S1 Text — Reaction time distribution for the three action regulation tasks. (DOCX) [file pcbi.1010111.s003.docx]

**S1 text**

We computed the distribution of the reaction times (RTs) for neurotypical participants and PD patients in the decision-making task, the Eriksen flanker task and the stop signal task (Fig. S1). Consistent with previous studies, we found that RT distributions are skewed to the right (i.e., positively skewed). This asymmetry tends to be more pronounced in PD patients (skewness range between [0.60 1.20]) than in neurotypical participants (skewness range between: [0.40 0.78]).
