# Supplementary material for: A neurocomputational theory of action regulation predicts motor behavior in neurotypical individuals and patients with Parkinson’s disease
Source: PLoS Comput Biol. 2022 Nov 17;18(11):e1010111. doi: 10.1371/journal.pcbi.1010111 (PMC9714880; doi:10.1371/journal.pcbi.1010111)
Supplement: S2 Text — We analyzed the reaching velocities in the three action regulation tasks. (DOCX) [file pcbi.1010111.s004.docx]

**S2 text**

We further explored motor behavioral differences between the two groups in the three action regulation tasks. We are interested in comparing the velocity of the reaching movements between neurotypical individuals and PD patients. To do so, we computed the maximum reaching velocity for both groups in the decision-making task, the Eriksen flanker task and the stop signal task. The velocity was calculated as the amount of distance (in this case, the axis value of the joystick) a joystick handle moves per second. In the decision-making task, we found that neurotypical individuals generated faster movements in both choice trials and instructed trials compared to PD patients (Fig. S2A, two-way ANOVA, p<0.001 for both choice trials and instructed trials). Interestingly, although the maximum velocity in instructed trials were significantly higher than that in choice trials for neurotypical individuals (Fig. S2A, two-way ANOVA, p<0.001) similar difference was not observed in PD patients (Fig. S2A, two-way ANOVA, p=0.3158). This may indicate that PD patients are moving so slow that they don’t need to further slow down in choice trials. Similar results were also observed in the Eriksen flanker task, where neurotypical individuals generated faster movements in both congruent and incongruent trials compared to PD patients (Fig. S2B, two-way ANOVA, p<0.001 for both congruent and incongruent trials), and the maximum velocity in congruent trials were significantly higher than that in congruent trials for neurotypical individuals but not PD patients (Fig. S2B, two-way ANOVA, p<0.01 for neurotypical individuals, p =0.6676 for PD patients). In the stop-signal task, notably, neurotypical individuals had higher maximum velocity compared to PD patients (Fig. S2C). In fact, although neurotypical individuals strategically delayed their responses in the stop signal task, in order to be more successful in stopping, they move faster than PD patients (two-tailed t-test p<0.001). We showed on previous study how the neurocomputational theory explains the effects of action competition to the velocity of the reaching movements [1]. In particular, the model predicts that averaging individual action plans produces slower movements that generating single actions plans towards a particular direction (for more information see the supplementary materials in [1]). This prediction is consistent with human findings from neurotypical individuals in which reaching movements performed in the presence of competing actions are slower than reaching movements instructed to a particular direction (see results from the current study and [1]). The model could also predict the slower movements of the PD patients compared to neurotypicals as a consequence of higher effort cost for performing reaching movements.

**References**

1. Enachescu V, Schrater P, Schaal S, and Christopoulos V. Action planning and control under uncertainty emerge through a desirability-driven competition between parallel encoding motor plans. *PLoS computational biology*, 17(10):e1009429, 2021.
