# Supplementary material for: A neurocomputational theory of action regulation predicts motor behavior in neurotypical individuals and patients with Parkinson’s disease
Source: PLoS Comput Biol. 2022 Nov 17;18(11):e1010111. doi: 10.1371/journal.pcbi.1010111 (PMC9714880; doi:10.1371/journal.pcbi.1010111)
Supplement: S1 Table — The values of the parameters of the neurodynamical framework. (PDF) [file pcbi.1010111.s005.pdf]

Model Parameters		
Parameters	Description	Value
η_{loc}	Visual input gain	8.5
η_{reward}	Expected outcome input gain	2.5
η_{cost}	Action cost input gain	-0.1
η_{pau}	Pause input gain	-4.0
γ	Action initiation threshold	0.6

Spatial sensory input field & Expected outcome field parameters		
Parameters	Description	Value
τ	Time constant	5.0
c_{exc}	Amplitude of excitatory portion of weight kernel	0
c_{inh}	Amplitude of inhibitory portion of weight kernel	0
σ_{exc}	Width of excitatory portion of weight kernel	5.0
σ_{inh}	Width of inhibitory portion of weight kernel	40.0
h	Resting activity level	-5.0
q	Noise level	0.25
σ_q	Width of noise kernel	5.0
β	Steepness of sigmoid activity function	1.0

Reach planning field parameters		
Parameters	Description	Value
τ	Time constant	5.0
c_{exc}	Amplitude of excitatory portion of weight kernel	0
c_{inh}	Amplitude of inhibitory portion of weight kernel	20
σ_{exc}	Width of excitatory portion of weight kernel	5.0
σ_{inh}	Width of inhibitory portion of weight kernel	180
h	Resting activity level	-5.0
q	Noise level	0.5
σ_q	Width of noise kernel	5.0
β	Steepness of sigmoid activity function	1.0

Pause field parameters		
Parameters	Description	Value
τ	Time constant	5.0
c_{exc}	Amplitude of excitatory portion of weight kernel	0
c_{inh}	Amplitude of inhibitory portion of weight kernel	0
σ_{exc}	Width of excitatory portion of weight kernel	5.0
σ_{inh}	Width of inhibitory portion of weight kernel	25.0
h	Resting activity level	-5.0
q	Noise level	0.25
σ_q	Width of noise kernel	5.0
β	Steepness of sigmoid activity function	1.0
